# Effects of a Goal-Oriented Intervention on Self-Management Behaviors and Self-Perceived Burden After Acute Stroke: A Randomized Controlled Trial

**DOI:** 10.3389/fneur.2021.650138

**Published:** 2021-07-20

**Authors:** Yu Chen, Yuanyuan Wei, Hongjuan Lang, Ting Xiao, Yan Hua, Lu Li, Jing Wang, Hongxia Guo, Chunping Ni

**Affiliations:** ^1^School of Nursing, Fourth Military Medical University, Xi'an, China; ^2^Leshan Retired Cadre Sanatorium, Leshan, China; ^3^College of Basic Medicne, Fourth Military Medical University, Xi'an, China; ^4^West China School of Nursing/West China Hospital, Sichuan University, Chengdu, China

**Keywords:** stroke, goal-oriented intervention, self-management behavior, self-perceived burden, randomized controlled trial

## Abstract

**Background:** Stroke generates significant health and social burdens. Self-management has potential importance for supporting individuals in coping and continuing to progress after stroke. However, there is a lack of targeted programs to enhance self-management and reduce self-perceived burden (SPB) following stroke.

**Purpose:** To evaluate the effects of a goal-oriented intervention on self-management behaviors and SPB among patients after acute stroke.

**Methods:** This was a randomized controlled trial with a 4-weeks intervention. Participants were randomly allocated to the intervention (*n* = 48) or control group (*n* = 48). The intervention and control groups received eight sessions of goal-oriented self-management intervention based on Pender's health promotion model and control care, respectively. Self-management behaviors and SPB were evaluated and compared between the two groups.

**Results:** After the 1-month follow-up, there were significant differences in the total self-management behaviors score and the scores of six of the self-management dimensions, excluding diet management, between the intervention group and the control group (*t* = −7.891– −2.815; *p* ≤ 0.006). Compared to the control group, the intervention group showed a significant decrease in the physical burden, emotional burden, and total SPB scores (*t* = 2.102–2.071; *p* = 0.015–0.041). The economic burden score was not significantly different between the two groups (*t* = 1.707; *p* = 0.091).

**Conclusion:** The goal-oriented intervention based on Pender's health promotion model can effectively improve self-management behaviors and reduce physical and emotional SPB among stroke survivors.

## Introduction

Globally, stroke was the third leading cause of disability-adjusted life-years (DALYs) and the second leading cause of death worldwide in 2017 (1, 2). In the United States, someone has a stroke approximately every 40 s, and someone dies of stroke every 4 min, and 50–70% of stroke survivors suffer from chronic neurological or cognitive impairment (3). The overall burden of stroke, including health, economic, and social costs, has been increasing for individuals, families, and national healthcare systems (1, 4). Stroke-related healthcare costs were estimated at $73.3 billion in 2010 (3). For stroke survivors, especially those with disabilities, receiving care imposes a high level of self-perceived burden (SPB). SPB is defined as “empathic concern engendered from the impact on others of one's illness and care needs, resulting in guilt, distress, feelings of responsibility, and diminished sense of self” (5). The study of Ren et al. showed that 65.8% of inpatients with stroke had SPB (6). SPB has negative influences on patients' rehabilitation and quality of life (7, 8). The mortality of recurrent stroke is higher than that of first-ever stroke (9–11). The negative impact of SPB will further increase the risk of stroke recurrence. Hence, stroke survivors' reduction in SPB should be considered equally important as other measures in their rehabilitation and the prevention of stroke recurrence.

Self-management has been advocated as one of the key strategies that enables individuals to reduce the risk of subsequent stroke, practice new healthy behaviors, and improve quality of life following stroke (12–14). At the national and international levels, self-management education programs have been recognized as an important approach to addressing the burden of chronic disease and helping individuals manage their condition more effectively (15, 16). However, evidence from six studies showed that self-management programs were not superior to other programs in terms of their effects in the domains of locus of control, activities of daily living, medication adherence, participation, or mood (17). In China, many stroke patients lack disease and rehabilitation management behaviors. There is little knowledge about the prevention of stroke recurrence, reduction in complications, and rehabilitation exercise (18). Individuals' capacity and support for self-management affect their stroke self-management (19). Currently, targeted programs to enhance self-management behaviors and reduce SPB following acute stroke are lacking.

Successful interventions for patients with chronic diseases should pay attention to health-promoting lifestyles and their influencing factors (20). Pender's health promotion model emphasizes assisting people in changing their lifestyles and moving toward a state of optimal health (21). Goal-oriented instructions were proven to be effective in increasing the intensity of practice in stroke rehabilitation (22). While previous studies have assessed health behavior modification after stroke, no randomized controlled trials have been developed to test the effectiveness and feasibility of an intervention combined with a self-management goal and Pender's model. Thus, the current study designed a goal-oriented self-management intervention based on Pender's health promotion model for stroke patients. Telephone follow-up intervention has been found to be effective in improving health behaviors among many chronic disease patients, such as hypertensive patients for cardiovascular disease risk reduction (23) and diabetes patients for self-care activities (24). In this study, the telephone follow-up intervention was delivered early after acute stroke. The main aim was to test whether the program could improve self-management behaviors and reduce the SPB of stroke patients in the first month after discharge.

## Methods

### Study Design

The study was a single-blind, randomized controlled trial with two parallel groups (1:1). Potential participants were recruited from the neurology department of a tertiary teaching hospital in Xi'an, China, from November 2017 to January 2018. If eligible, patients formally consented to participate in the study. Data collection at baseline and postintervention was completed on the fourth day postadmission (T1) and at the time of the 1-month outpatient review (T2), respectively. The measures were self-administered by the patients. For the patients with visual impairment or low education level, the data coordinator read the items for them and filled in the questionnaire after they answered. This randomized-controlled trial was conducted in accordance with the Declaration of Helsinki, and was registered with ClinicalTrials, the registration number is ChiCTR200040805.

### Inclusion and Exclusion Criteria

The inclusion criteria were (a) age of 18 years or older and (b) diagnosis of acute stroke by computed tomography or magnetic resonance imaging. Patients with serious complications such as the following were excluded: severe heart, liver, and kidney diseases; reduced level of consciousness; cognitive disabilities; psychosis; or deafness, aphasia, or other language barriers.

### Randomization

The random allocation sequence was computer generated by an independent researcher who did not know the identities of the patients and had no contact with them. Group allocation was performed by sending sealed envelopes with a serial admission number on the outside and a sheet of paper inside with the group name to the study coordinator. The coordinator enrolled the patients and assigned them to different groups according to the sheet of paper inside the envelope.

### Interventions

On the discharge day, all participants received discharge education and a video disk of rehabilitation exercises for postdischarge training. In the first month after discharge, the participants in the intervention and control groups received eight sessions of goal-oriented self-management intervention and control care, respectively.

The goal-oriented self-management intervention was designed as a one-to-one telephone session delivered twice weekly for ~30 min. The intervention was developed based on Pender's health promotion model (25). Pender's model includes six dimensions of health-promoting lifestyles: physical activity, nutrition, stress management, health responsibility, interpersonal support, and self-actualization (21). The goals and content of the intervention focused on these six dimensions. In addition, stroke medication, stroke monitoring, and secondary prevention were provided (Table 1). The intervention was delivered by telephone follow-up. During each telephone call, the participants' autonomy and healthy behaviors were promoted through five processes: perceiving problems, expressing emotions, adjusting goals and plans, and receiving targeted education and evaluation (21).

**Table 1:** Contents and goals of the goal-oriented self-management intervention.

**Session**	**Intervention theme**	**Goals**
Session 1 (1st week)	Health responsibility - Importance of self-management - Smoking - Alcohol intake	• To stimulate motivation and autonomy for behavioral change • To stop smoking • To drink no more than 25 g/days of pure alcohol for male, 15 g for female
Session 2 (1st week)	Stroke monitoring and recurrence prevention - Stroke warning symptoms - Stroke sequel and complications - Stroke prevention	• To understand stroke facts and prevention of stroke
Session 3 (2nd week)	Stroke medication - Correct usage of medicine - Precautions for medicine using - Adverse reactions of medicine	• To take medicine as prescribed
Session 4 (2nd week)	Nutrition - Importance of daily diet in promoting stroke recovery and recurrence prevention - Reasonable diet - Precautions for daily diet	• To have a reasonable and balanced diet • To maintain a low salt intake <6 g/days
Session 5 (3rd week)	Physical activity/ rehabilitation exercise - Method, frequency and intensity of exercise - Precautions for exercise	• To have a positive attitude on physical activity/rehabilitation exercise • To have moderate to intense exercise 3–5 days/weeks for over 30 min/days
Session 6 (3rd week)	Stress management - Stressors after stroke - Barriers to stress management - Skills to cope with stress	• To establish the confidence to rehabilitation • To cope with stress properly
Session 7 (4th week)	Interpersonal support - Importance of interpersonal support - Interpersonal resources - Skills to get interpersonal support	• To describe potential support from society and family • To have a good interpersonal support
Session 8 (4th week)	Self-actualization - Self-management after stroke - Barriers to self-actualization - Adjustment after stroke	• To maintain motivation to self-management • To cope with negative influences of stroke on daily living and work

The main strategy for the control care was health education. Within 4 weeks after discharge, the participants in the control group received telephone follow-up twice weekly for ~30 min each session. At each follow-up, the education targeted providing responses to the participants' questions. The content of the health education could involve medication, diet, rehabilitation exercise, and the prevention of recurrent stroke.

### Blinding

The patients were blind to their group assignments. The interventions were undertaken by two experienced neurology nurses who were not blind to the group allocations. The outcomes before and after the intervention were assessed by a graduate-level nursing student unaware of the group allocation. Data entry and analysis were conducted by an individual who was blind to the group allocation.

### Baseline Data

The participants completed questionnaires including items on demographic information (age, gender, marital status, and educational level), medical expenses, self-evaluated economic pressure and disease knowledge, stroke (number and types of strokes), and level of physical disability.

The level of physical disability was measured using the Barthel Index (BI). The BI has 10 items, with 2 items scored from 0 to 15, six scored from 0 to 10, and two scored from 0 to 5. The total score ranges from 0 (total dependence) to 100 (total independence). A higher BI indicates a higher level of activities of daily living (ADL) ability (26). For better interpretation, the results were grouped into four categories: severe disability (BI, ≤40), moderate disability (BI, 41–60), mild disability (BI, 61–99), and no disability (BI = 100). The BI is widely used among Chinese stroke patients (27). The Cronbach's alpha was 0.88 in this study.

Disease knowledge was assessed by asking how much participants knew about stroke. The level of knowledge was divided into three levels: rich, normal, and deficit.

### Outcome Measures

The primary outcome was self-management behaviors, which were assessed by the Stroke Self-management Scale (SSMS). The SSMS was designed by Wang et al. (28). The 50-item scale was validated and found to have satisfactory reliability and validity among stroke patients (Cronbach's alpha = 0.84, content validity = 0.95). The SSMS is composed of seven subscales: symptom and sign monitoring, medication, diet, daily life, emotion, rehabilitation exercise, and social function and interpersonal relationship management. Each item is rated on a five-point Likert scale (1 = no to 5 = always). Higher scores represent better self-management behavior.

The secondary outcome was SPB, which was assessed by the Chinese version of the Self-Perceived Burden Scale (SPBS). The SPBS was originally developed by Cousineau et al. (29). It is a 10-item self-report measurement that includes three subscales: physical burden, emotional burden, and economic burden. Each item is rated on a 5-point Likert scale to indicate the degree of SPB. Higher scores indicate higher levels of SPB. According to the total score, SPB was divided into three levels. Scores in the ranges of 20–30, 30–40, and ≥40 were considered to indicate mild, moderate, and severe SPB, respectively. The scale has acceptable internal consistency, with a Cronbach's alpha of 0.91 (30).

### Ethics Approval

This randomized controlled trial was registered with ClinicalTrials, and the registration number is chiCTR2000040805 (http://www.chictr.org.cn/showproj.aspx?proj=63248). Approval for the study was granted by the Human Research Ethics Committee of Xi'an Jiaotong University Health Science Center (No. 2017926). Informed consent was obtained from each participant.

### Sample Size

The sample size was estimated based on the formula for clinical trials as follows: *n* = 2σ^2^ × f(α, β)/(μ_1_ − μ_2_)^2^. According to the pilot study, σ = 12.41, α = 0.05, β = 0.10, μ_1_ = 1.96, μ_2_ = 1.28, and n_1_ and n_2_ were 40, as calculated by the formula. Allowing for 20% attrition, the sample size was calculated to be 48 per group.

### Statistical Analysis

SPSS 19.0 software was used to perform statistical analysis, and *p* < 0.05 was used to establish statistical significance in all comparisons between groups. Descriptive analysis was conducted using the percentage and frequency for categorical variables and the mean and standard deviation for continuous variables. Continuous variables were compared using Student's *t*-test, and categorical data were compared using the chi-square test. Independent *t*-tests were used to compare the di?erences between the intervention and control groups. Paired *t*-tests were performed for comparisons within groups between the two time points.

## Results

### Baseline Characteristics of the Participants

**Figure 1:**
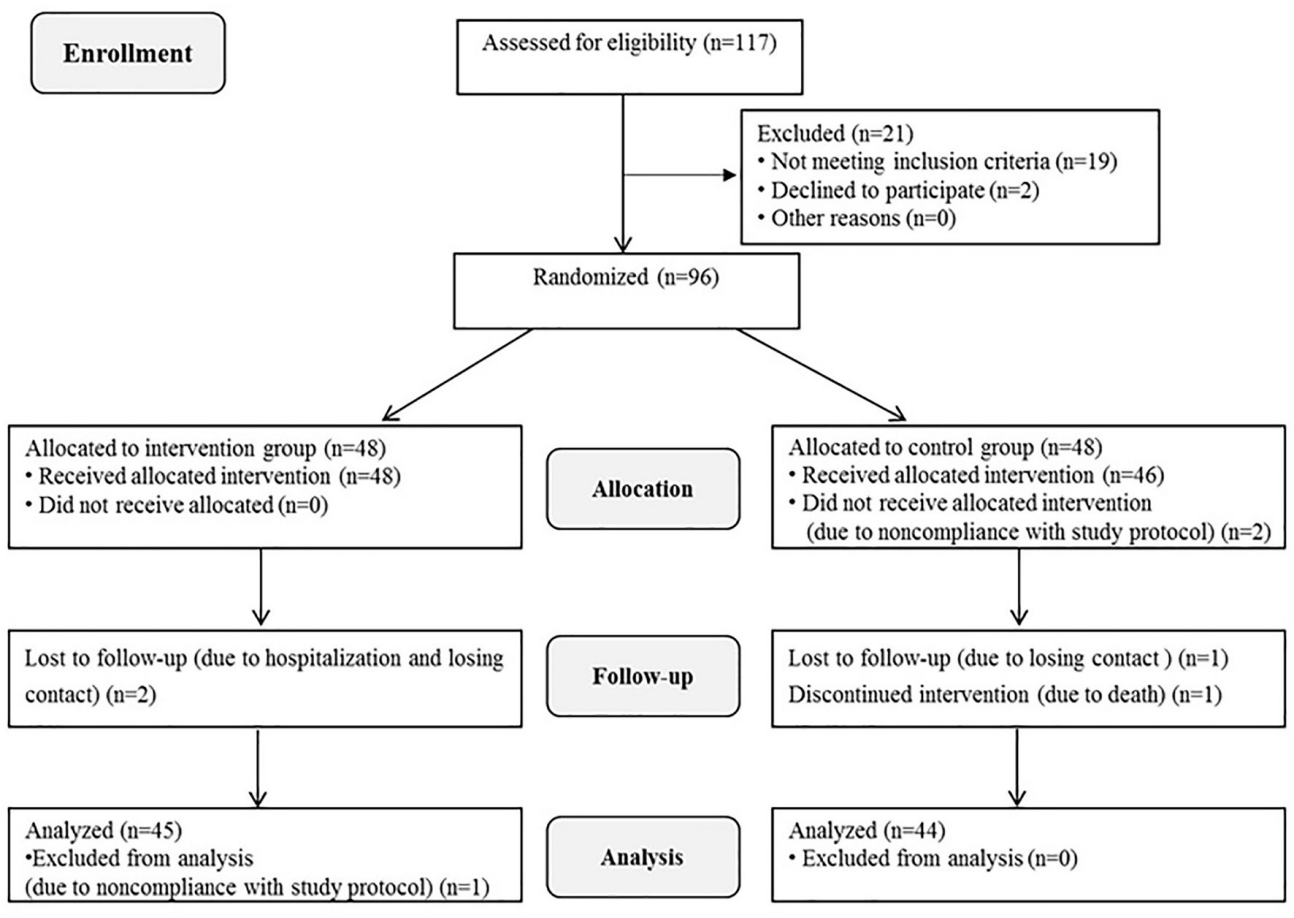
Consort participant flow diagram.

A total of 96 patients were enrolled in the study. After some patients were excluded due to withdrawal, loss to follow-up, and death, 45 patients in the intervention group and 44 in the control group were included in the final data analysis. The specific reasons for loss to follow-up are illustrated in Figure 1. The mean age of the participants was 61.91 years, and their age range was 42–83 years. Eighty-seven patients (97.7%) suffered from ADL disability. There were no significant differences in general information between the two groups at baseline (*p* = 0.264–1.000). The characteristics of the participants are presented in Table 2.

**Table 2:** Characteristics of participants at baseline (*n*, %).

	**Intervention (*n =* 45)**	**Control (*n =* 44)**	**t/χ^2^**	***p***
Age (years)	63.11 ± 10.73	60.68 ± 9.63	1.123^a^	0.264
Gender, *n* (%)			0.029^b^	0.865
Male	32 (71.1)	32 (72.7)		
Female	13 (28.9)	12 (27.3)		
Marital status, *n* (%)			0.000^b^	1.000
Having a spouse	43 (95.6)	43 (97.7)		
No spouse	2 (4.4)	1 (2.3)		
Educational level, *n* (%)			1.970^b^	0.579
Elementary school	11 (24.4)	6 (13.6)		
Junior high school	14 (31.1)	17 (38.6)		
Senior high school	13 (28.9)	5 (11.4)		
College or above	7 (15.6)	6 (13.6)		
Medical expenses, *n* (%)			2.001^b^	0.157
Medical insurance	43 (95.6)	44 (100)		
At one's own expense	2 (4.4)	0 (0.0)		
Self-evaluated economic pressure, *n* (%)			0.014^b^	1.000
Low	20 (44.4)	19 (43.2)		
Moderate	13 (28.9)	13 (29.5)		
High	12 (26.7)	12 (27.3)		
Physical disability, *n* (%)			1.503^b^	0.785
Severe disability	8 (17.8)	9 (20.5)		
Moderate disability	27 (60)	29 (65.9)		
Mild disability	9 (20)	5 (11.4)		
Independence	1 (2.2)	1 (2.3)		
Number of stroke, *n* (%)				
1	27 (60)	29 (65.9)		
≥2	18 (40)	15 (34.1)		
Self-evaluated disease knowledge, *n* (%)			0.023^b^	0.879
Rich	0 (0.0)	0 (0.0)		
Normal	15 (33.3)	14 (31.8)		
Deficit	30 (66.7)	30 (68.2)		
Type of stroke, *n* (%)			0.000^b^	1.000
Hemorrhagic stroke	5 (11.1)	4 (9.1)		
Ischemic stroke	40 (88.9)	40 (90.9)		

a *t-value*.

b *Chi-square value*.

### SSMS and SPBS Scores in the Two Groups

**Figure 2:**
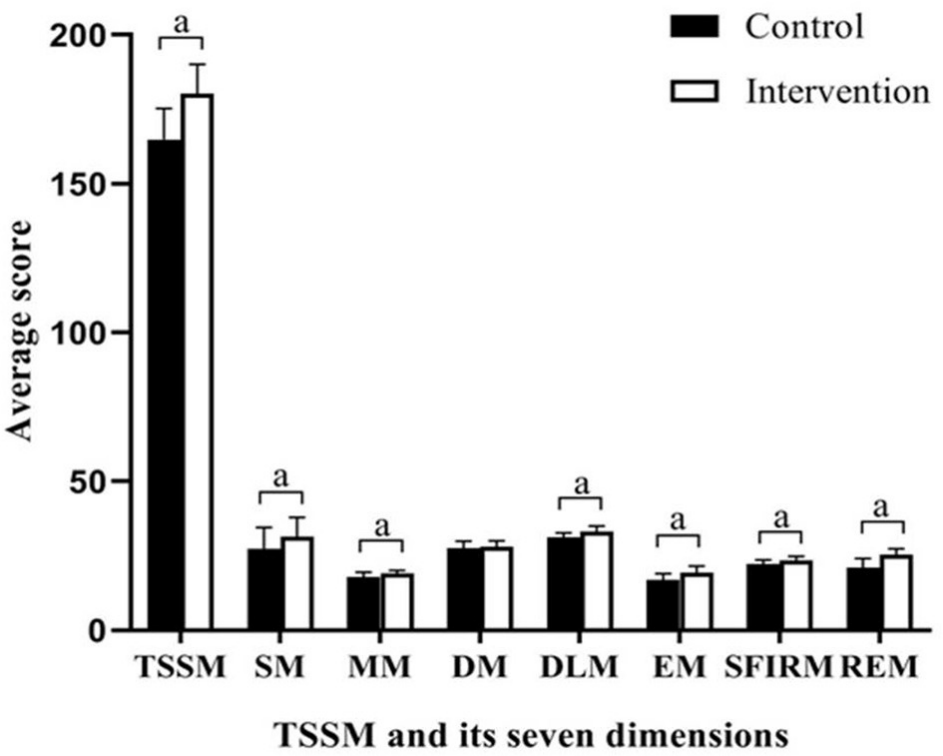
Differences between groups in scores of SSMS at completion of the intervention. SSMA, stroke self-management scale; SM, symptoms and sign monitoring; MM, medication management; DM, diet management; DLM, daily life management; EM, emotion management; SFIRM, social function and interpersonal relationship management; REM, rehabilitation exercise management; TSSM, total stroke self-management; ^*a*^*P* < 0.01.

The primary outcome, SSMS scores, is presented in Table 3 and Figure 2. There were no significant differences in the total SSMS scores or the seven dimension scores at T1 between the control and intervention groups. Both groups showed improvements in the total SSMS score and six of the seven dimension scores, excluding the reduction in the emotion management score in the control group, over time. The total SSMS score and six of the dimension scores, excluding the diet self-management dimension score, in the intervention group were significantly higher than those in the control group at T2 (*t* = −7.891–−2.815; *p* ≤ 0.006).

**Table 3:** Comparison of scores for SSMS between two groups.

**SSM**	**Control group (*n =* 44)**					**Intervention group (*n =* 45)**					**Between groups**	
	**T1**		**T2**		**T1 control vs. T2 control**	**T1**		**T2**		**T1 intervention vs. T2 intervention**	**T1 control vs. T1 intervention**	**T2 control vs. T2 intervention**
	**Mean**	**SD**	**Mean**	**SD**	***t***	**Mean**	**SD**	**Mean**	**SD**	***t***	***T***	***t***
SM	24.82	7.03	27.45	7.11	−8.739^**^	23.80	6.20	31.49	6.40	−24.094^**^	0.726	−2.815^*^
MM	13.86	5.86	17.89	1.60	−4.702^**^	14.27	5.40	19.22	0.95	−6.042^**^	−0.338	−4.795^**^
DM	25.82	2.42	27.68	2.30	−7.132^**^	25.16	1.86	28.09	1.99	−13.698^**^	1.450	−0.894
DLM	29.14	2.14	31.32	1.44	−7.634^**^	28.84	1.77	33.09	1.88	−15.204^**^	0.702	−4.975^**^
EM	18.84	1.41	17.05	2.06	7.407^**^	18.49	1.24	19.36	2.20	−4.280^**^	1.252	−5.119^**^
SFIRM	20.77	1.34	22.36	1.33	−7.039^**^	20.33	1.04	23.53	1.31	−16.420^**^	1.724	−4.182^**^
REM	15.68	3.02	21.07	3.08	−13.941^**^	15.47	3.03	25.42	1.99	−26.478^**^	0.336	−7.891^**^
TSSM	148.91	10.78	164.82	10.39	−11.841^**^	146.36	9.38	180.20	9.88	−26.962^**^	1.193	−7.156^**^

*T1, at baseline; T2, at completion of the intervention; SSMS, stroke self-management scale; SM, symptoms and signs monitoring; MM, medication management; DM, diet management; DLM, daily life management; EM, emotion management; SFIRM, social function and interpersonal relationship management; REM, rehabilitation exercise management; TSSM, total stroke self-management; mean and SD, mean score and standard error (SD) for SSMS*.

* *p = 0.006;*

** *p < 0.001*.

**Figure 3:**
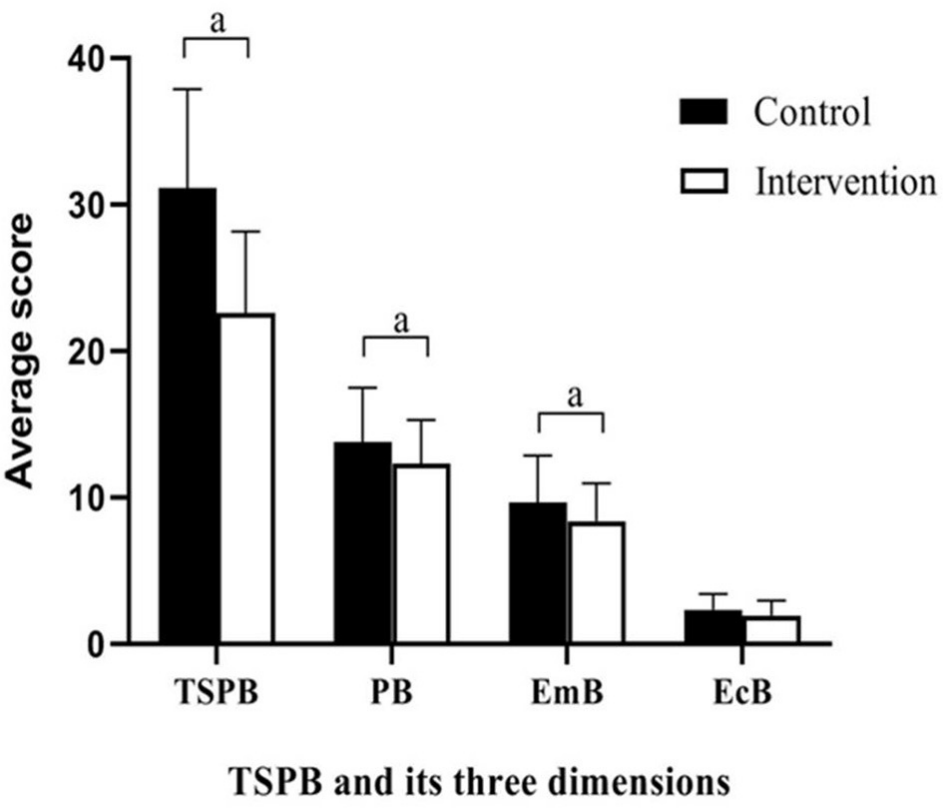
Differences between groups in scores of SPBS at completion of the intervention. Emb, emotional burden; EcB, economic burden; SPB, self-perceived burden; TSPB, total self-perceived burden; ^*a*^*P* < 0.05.

The secondary outcome, SPBS scores, is shown in Table 4 and Figure 3. There was no significant group difference in the baseline scores. Compared to baseline, after the intervention, the SPBS scores all decreased in the two groups. The intervention group showed significantly larger decreases than the control group in the physical burden, emotional burden, and total SPBS scores (*t* = 2.102–2.071; *p* = 0.015–0.041). The economic burden score was not significantly different between the two groups (*t* = 1.707; *p* = 0.091).

**Table 4:** Comparison of scores for SPBS between two groups.

**SPB**	**Control group (*n =* 44)**					**Intervention group (*n =* 45)**					**Between groups**	
	**T1**		**T2**		**T1 control vs. T2 control**	**T1**		**T2**		**T1 intervention vs. T2 intervention**	**T1 control vs. T1 intervention**	**T2 control vs. T2 intervention**
	**Mean**	**SD**	**Mean**	**SD**	***t***	**Mean**	**SD**	**Mean**	**SD**	***t***	***t***	***t***
PB	15.34	3.89	13.80	3.68	6.165^**^	15.60	3.65	12.33	2.95	10.362^**^	−0.324	2.071^*^
EmB	12.36	3.04	9.66	3.21	8.336^**^	12.53	3.03	8.36	2.62	17.841^**^	−0.263	2.102^*^
EcB	3.05	1.61	2.32	1.09	5.368^**^	2.98	1.57	1.93	1.03	7.548^**^	0.200	1.707
TSPB	30.75	6.92	31.11	6.75	10.040^**^	31.11	6.75	22.60	5.57	17.634^**^	−0.250	2.470^*^

*T1, at baseline; T2, at completion of the intervention; PB, physical burden; EmB, emotional burden; EcB, economic burden; SPB, self-perceived burden; TSPB, total self-perceived burden; mean and SD, mean score and standard error (SD)*.

* *p < 0.05;*

** *P < 0.001*.

## Discussion

This study found that telephone follow-up was an efficient approach to improving health behaviors among stroke patients, which is consistent with a previous study on survivors of colorectal cancer (31). In the control and intervention groups, there were significant changes in patients' self-management of stroke symptoms and sign monitoring, medication, daily life, emotion, rehabilitation exercise, social function, and interpersonal relationships. Different from previous studies (17), the goal-oriented self-management intervention was found to be effective in the activities of daily living and medication adherence. Of these changes, the rehabilitation dimension changed the most. This finding may be explained by the flexible strategies that the goal-oriented self-management intervention provided. Unlike previous programs (32, 33), the goal-oriented self-management intervention was based on Pender's health promotion model. Pender's health promotion model is an important tool to motivate patients with chronic diseases to adopt or maintain healthy lifestyle behaviors (34). The aim of this intervention was to stimulate motivation and behavior changes to promote rehabilitation and prevent recurrent stroke. The overall goal comprised a series of subgoals corresponding to the follow-up sessions. At each follow-up, the patients interacted with healthcare professionals to engage in shared decision-making and resolve life-disease conflict to facilitate their adjustment to the complex, dynamic situation after stroke. As a result, patients' motivation for self-management could be stimulated, and their unhealthy behaviors could be changed.

It should be mentioned that there was no significant difference in the dietary self-management changes between the two groups in this study. A possible reason for this finding is that eating habits are a regular behavior formed over the long term in one's life, and they are thus difficult to change in a short time (35). The study also found that the level of emotion self-management in the control group did not improve but was significantly reduced after 1 month of control care. A possible reason for this finding is that the control care paid more attention to knowledge acquisition and behavior modification but ignored emotional expression and counseling. The results indicate that it is important to improve patients' mood following acute stroke.

In this study, both control care and goal-oriented intervention were proven to be effective in reducing physical and emotional burdens at an early stage after acute stroke, and the goal-oriented intervention decreased patients' SPB to a great extent. The significantly decreased SPB in the intervention group may be explained by their goal setting. Goal setting is a process in which people set targets and work toward achieving them (36). It is recognized as a potentially effective technique for assisting patients in adopting healthier behaviors (37). The improvement of health behavior promotes patients' recovery and reduces physical and emotional burdens. In addition, there was no significant difference in patients' economic burden between the two groups after the intervention. The possible reason is that patients after stroke need long-term sequelae treatment and secondary prevention, which will increase their economic burden (38). The results indicate that intervention in stroke survivors' SPB is a long-term process and that the reduction in economic burden requires the joint efforts of medical staff, patients, and their families.

The results of this rigorous randomized controlled trial indicate the efficacy of early intervention following acute stroke; however, there are some limitations in this study. The participants were recruited from a general hospital in one city, and it is unclear whether these results are generalizable beyond the study population to other similar populations. According to the inclusion/exclusion criteria, stroke patients with aphasia or cognitive impairment were excluded. It was both critical and necessary for all participants to improve their self-management behaviors and reduce their SPB following stroke. Nonetheless, even participants with severe language impairment or mild cognitive impairment were able to initiate and complete their intervention programs once the education and training were provided. Further research should adjust the recruitment approach to benefit more patients with stroke. Additionally, process data were not collected during the 1-month intervention, and the intervention was relatively short, which might limit the understanding of the findings. In the future, multicenter studies are needed to extend the follow-up period and to assess the process and long-term effects of the intervention.

While more studies are needed, this study has some practical implications. The findings provide evidence of the effectiveness of self-management intervention programs. In this study, a goal-oriented intervention based on Pender's health promotion model was developed and implemented for patients following acute stroke. The goal-oriented strategy is practical for stroke survivors to confront the increasing complexity of postdischarge rehabilitation and recurrence prevention at the early stage of stroke. The achievement of goals is beneficial to the improvement of self-management behaviors and the reduction in SPB among stroke survivors. To promote recovery from stroke, patients, caregivers, and nurses should be aware of the importance of goal-oriented strategies in enhancing self-management following stroke.

## Conclusion

Goal-oriented intervention based on Pender's health promotion model can effectively improve survivors' self-management behaviors and reduce the level of SPB at the early stage of acute stroke. The findings extend our knowledge of advanced nursing practice for delivering goal-oriented self-management interventions to meet the complex and dynamic needs of patients with stroke. The study provides a reference for transitional care for other chronic diseases.

## Data Availability Statement

The raw data supporting the conclusions of this article will be made available by the authors, without undue reservation.

## Ethics Statement

The studies involving human participants were reviewed and approved by the Human Research Ethics Committee of Xi'an Jiaotong University Health Science Center. The patients/participants provided their written informed consent to participate in this study.

## Author Contributions

YC and YW: conceptualization, data curation, and writing-original draft. HL, TX, and YH: methodology and data curation. LL and JW: formal analysis. CN and HG: conceptualization, methodology, funding acquisition, and writing—review and editing. All authors contributed to the article and approved the submitted version.

## Conflict of Interest

The authors declare that the research was conducted in the absence of any commercial or financial relationships that could be construed as a potential conflict of interest.
